# Hospital and Institutional News

**Published:** 1919-10-18

**Authors:** 


### HOSPITAL AND INSTITUTIONAL NEWS.

### MUSEUM DEMONSTRATIONS.

At the Royal College of Surgeons of England, the following demonstrations of Diseases and Disorders following lesions of the Glands of Internal Secretion will be given in the Theatre of the College in Lincoln's Inn Fields by Professor Arthur Keith, the Conservator of the Museum. These demonstrations are open to medical students and practitioners. First-aid and ambulance students desirous of attending will also be admitted. Friday, October 24, at 5 P.M. Acromegaly; Friday, October 31, 5 P.M., Achondroplasia; Friday, November 7, Multiple Exostoseo.

### THE MILK SHORTAGE.

A conference, under the auspices of the Association of Medical Officers of Infant Welfare and Maternity Centres will be held on Friday, October 24, at 6 P.M., at Bedford College for Women, Regent's Park, N.W., to discuss the feeding of infants and young children in view of the shortage and high price of cow's milk, and with special reference to the use of dried milk and patent foods. Admission is free and no tickets are required.

### THE MEDICAL EXHIBITION.

The ninth London Medical Exhibition was opened at the Central Hall, Westminster, on October 6, and remained open until Friday, October 10. Although the Horticultural Hall, where the previous exhibitions were held, certainly gives more space, the position of the Central Hall is greatly in its favour as a venue for this function, and the change has apparently worked quite satisfactorily. As was to be expected, the features of more particular interest were those showing the wonderful advances made in the manufacture of chemical and pharmaceutical products. It has, of course, been a direct effect of war conditions, and manufacturers are to be congratulated both upon the success of their endeavours to replace imported units and upon the fact that the majority now appear to be established on a scale which guarantees adequate and reliable home supplies of any drugs hitherto obtainable only from abroad. As usual, the organisers made a point of keeping the exhibition exclusive to the medical visitor, and the advantage of being able thus to study at leisure a centralised and beautifully arranged demonstration of so many new things in medicine and surgery has been keenly appreciated by the profession. The drug, surgical, and scientific instrument and book trades were most excellently represented, but, as already explained, the main object of this year's exhibition has been to illustrate the success of the British manufacturer in replacing wares formerly of foreign origin only. The result was that the visitor could not fail immediately to be impressed with the ability that British men of science have demonstrated in a wonderfully successful attempt to break the monopoly of enemy enterprise. May such progress continue. We have every confidence that it will, and that the medical exhibition of next year will show yet more rapid advances. In any event, the interest of this excellent demonstration more than repaid a visit.

### CRIPPLED CHILDREN.

When put forward by so great an orthopædic surgeon as Sir Robert Jones, a suggestion as to the means by which we may markedly improve the percentage of cures among crippled children is well worthy of public notice. Writing in the *British Medical Journal*, Sir Robert gives a detailed account of the measures which he proposes. In the first place, rickets and tuberculosis account between them for 40% of the crippled children in an average locality. These particular diseases are preventable, but the causes of the remaining 60% are at present so ill-defined that we cannot justifiably hope for extensive prevention. For example, we are impotent to prevent congenital conditions and are almost without power to eliminate many early paralyses. We know by experience that although many die, a large number of these children grow up as more or less hopeless cripples. Yet, as the author emphasises, many are fully curable, but up to the present the nation has made but little united effort to face the problem. He now proposes to unite the duty of caring for these children to those of the orthopædic hospitals already under the control of the Ministry of Pensions. The organisation is there and in full working order, it is simply extension which must be made. Each district must have its open-air country orthopædic hospital and an out-patient clinic system. The specially-trained doctors and nurses are already in being as a post-war consequence, and it is proposed that the Ministry of Health should take action in the matter and make provisions in advance, so that, as the actual pensions work diminishes, the staffs may not be unwisely dispersed.

### ARTIFICIAL LIMBS.

An Army Council Instruction just issued provides that an officer who requires an artificial limb in consequence of having been wounded in action or injured in the performance of military duty may either obtain it under Article 647 of the Pay Warrant, or be provided with it at a special "fitting" hospital. As explained by the *Times*, an officer who continues to serve may be supplied with a spare or duplicate limb under the same conditions. The scale up to which grants under the Pay Warrant may be made is as follows: For hip amputation, £30; ordinary leg, with pelvic band, £27 15s.; ordinary leg, without pelvic band, £24 15s.; for Symes' amputation, £15 15s.; arms, from £10 to £20; mechanical arms (when required for the officer's future profession, £40. Officers who avail themselves of a grant under the Pay Warrant or desire information regarding the "fitting" hospitals should apply to the Registrar, Central Registry for Limbless Sailors and Soldiers, Cromwell House, Millbank, London, S.W. 1. The special "fitting" hospitals at which officers may attend either as in-patients or out-patients are Queen Mary's Convalescent Auxiliary Hospital, Dover House, Roehampton, S.W. 15; 5th London T.F. General Hospital (St. Thomas's), Lambeth, S.E.; the Prince of Wales' Hospital, The Walk, Cardiff; Edenhall Limbless Hospital, Musselburgh, N.B. (out-patients only); Ulster Volunteer Force Hospital, Belfast, Ireland.

### THE PERIL OF TYPHUS.

The League of Red Cross Societies in Geneva urge the necessity of taking the strongest measures for preventing the possible outbreak of epidemics of typhus in Western Europe. There is apparently serious risk that the disease will thus re-occur in Poland this winter, and great efforts are needed, not only to save the inhabitants, but also in the interest of Western Europe and America. Such is the summary of the conditions found by the Medical Commission sent to investigate the situation, and including four men expert in controlling epidemic disease—namely, Colonel Hugh S. Cumming, Assistant Surgeon to the United States Health Service (Chairman), Colonel G. S. Buchanan (Senior Medical Officer of the British Ministry of Health), Colonel A. Castellani (Italian Medical Service), and Colonel J. Vesbecq (Médécin Principal of the French Army). The most urgent need is for soap, underclothing, outerclothing, blankets, hospital equipment and utensils. Drugs are also wanting, especially salvarsan, iodides, quinine, castor oil, salol and opium. There are many hospitals without beds, sheets or blankets, and at least fifty additional doctors and one hundred nurses are absolutely necessary.

### VENEREAL PREVENTION.

It is but a short time ago that upon the wisdom of findings by the National Council for Combating Venereal Disease there was so great and active a disagreement not only among the general public but even among the purely medical journals themselves that, apparently as the result of this unsatisfactory situation, the Venereal Prevention Committee came into being. An echo of the divergent policies of these two bodies was found in the appearance in the *Times* of October 6 of a letter from the Council, and, two days later, of one from Sir Bryan Donkin, a leading member of the Committee. The second letter, as the *Times* pointed out, made the present position quite clear to any unprejudiced reader, and the inference to be drawn from the reading of both is that the issue has been sufficiently narrowed, not strictly perhaps to a quibble on the relative values of the stearate and the permanganate of potassium as cleansing agents and bactericides, but at least to the finer adjustment of exact means by which local prevention can with satisfactory discretion be introduced. Truly does the public " stand to gain by the fact that both Council and Committee are now agreed that prevention is better than cure."

### "TUBERCLE."

The new monthly journal with the above title, the prospectus of which we noticed some months back, has now made its appearance to time, in spite of the difficulties which the railway strike must have caused. The first number has impressed us very favourably. It is designed with a good service of abstracts of foreign literature, and other features rendering it eminently useful to the tuberculosis worker, lay as well as professional. The reading matter proper is not sacrificed to the advertisements, and there are no puffs of proprietary articles, while the paper has a matt surface without those violent contrasts of type which blind like the headlights of a motor-car. The original articles begin with one by a Norwegian, on a recent development of lung surgery; a second touches, rather superfluously perhaps, the well-worn subject of the classification of phthisis; while Sir St. Clair Thomson contributes an interesting case of prolonged latency of tuberculosis. Altogether, this journal should raise England's position in the matter of tuberculosis journalism.

### NURSING SCHEME FOR THE INSURED.

The Lambeth Medical Officer of Health recently drew the attention of the London Insurance Committee to difficulties experienced by insured persons suffering from influenza in obtaining nursing attention. Insurance Committees have power to support nursing associations. When the London Committee went into the matter, it came to the conclusion that the best course to adopt would be not to attempt to set up a nursing scheme of its own, but rather to subscribe to established nursing associations with a view to securing the provision of nursing services for insured persons. To this end it communicated with the Central Council for District Nursing in London, which declared that £1,000 would by no means cover the annual cost, but promised that, pending other and more permanent arrangements, to allocate amongst the various nursing associations a contribution of this kind from the Insurance Committee, which would be of great assistance in promoting the supply of nurses in London, and would secure that no insured person would be left without nursing attention. The Insurance Committee, however, discovered that its finances prohibited it from making a substantial grant, but intimates that the question of nursing services will be reconsidered at a future date.

### A NEW WELSH MATERNITY HOSPITAL.

The recent announcement that Major J. W. Benyon, J.P., the county director of the Red Cross in Monmouthshire, has purchased Troedrhiw-Fawr, Newbridge, to present the County Council and the County Nursing Association with a maternity hospital, is welcome news. It is only a year since Major Benyon presented a large house north of Newport to the American Y.M.C.A. for conversion into a naval hospital. He has also successfully pressed the claims of Monmouthshire institutions for participation in the grants lately made from the surplus funds in the hands of the joint societies. Since Major Benyon is understood to have made himself responsible for the equipment of the house at Newbridge, and for the building of a new theatre there, his gift is very generous. When we hear of any new hospital in South Wales we always hope that, directly or indirectly, one of its effects will be to lighten the immense burden of work which now weighs upon King Edward VII. Hospital, Cardiff. The long waiting-list there, and the heavy overdraft, need in Cardiff men like Major Benyon, with ideas and the means to carry them out. Cardiff seems to suffer from the presence of men of means without ideas; for though it has raised a large sum, the sum is a trifle compared to the immense wealth of the city.

### THE MEDICAL DEFENCE UNION.

Once more this valuable society is to be congratulated on a fine record of work done, as disclosed in the Annual Report for 1918 recently issued, and a further strengthening of its prestige and financial resources. To the loss which the Union has sustained in the death of the general secretary, Dr. Bateman, the report makes feeling reference; and it is fortunate that for a post which makes very peculiar demands, the Union has been able to secure a successor well qualified to discharge them. It has been a custom with occasional teachers at the medical schools to impress on students every now and then the vital importance of their joining a medical defence society as soon as they shall be qualified and registered. But this wise custom is far too restricted, for experience shows that the majority of newly-qualified men (during the war years at any rate) had either never heard of the Defence Union, or confused it with the British Medical Association, or regarded it as a rival body endeavouring to supplant the latter. It is partly to correct such delusions, and to disseminate a wider knowledge of the beneficent activities of the Medical Defence Union, that its Council convenes the annual meeting in different centres, *e.g.*, this year at Leicester; a plan for which there is much to be said, provided that London, as the most generally accessible and amenable locality, is selected not less often than, say, one year in four. Meanwhile it is satisfactory to know that the Union's career of prosperity and usefulness continues unchecked: the Report mentions that no case taken to Court has been lost in the last two years.

### THE DUBLIN HOSPITAL'S NEW START.

A climax of misfortune is sometimes a blessing in disguise. This seems to be true of the Richmond, Whitworth, and Hardwick Hospitals, Dublin, which lately declared themselves unable to continue, shut down, and appealed in vain for an increase of the Government grant. Mr. W. Webster Smith, the Secretary, whose committee hoped to resume work in October, announced that the hospitals intend to surmount the refusal of the Treasury to increase its grant by adopting Lord Crawford's suggestion, which was made in the Upper House on August 13, namely, to collect weekly payments from patients who can afford to contribute. In fact, some attempt to cater for paying patients seems to be intended, though no necessitous case will be refused, except in the absence of a bed. The clinical teaching of students will be resumed, and some sort of appeal is being made to the general public.

### THE MEMOIRS OF A POOR-LAW CLERK?

Since Dickens exposed their weaknesses, improvement has occurred in the methods and men who are responsible for the administration of the Poor Law. For this reason we wish that Mr. P. Harding Roberts, who has resigned after thirty-two years work as clerk to the Holywell Board of Guardians, would write his memoirs. Since 1887 the changes have been numerous, and the impressions of a man who lived through them and had, indeed, to carry them out, would be interesting, especially if, as may be the case, he could bring forward unexpected instances to show that some changes have not been improvements. In recognition of Mr. Harding Roberts' work, the chairman of the Board, Mr. James Price, proposes to move that eight years be added to the clerk's term of service, so that the maximum period of forty years may be made the basis on which Mr. Roberts' superannuation shall be reckoned. After twelve months' leave of absence in consequence of ill-health, Mr. Roberts' long service should be generously recognised.

### WORKMEN'S CONVALESCENT HOMES.

The Leeds Workpeople's Hospital Fund has decided to purchase the Crossbeck Convalescent Home, Ilkley, which the Fund has rented for the past six years. It accommodates twenty-four patients; its price is £2,750. This is another instance of the useful ramifications of these funds, which began by collecting pennies, and have become the centre of activities and institutions with a manifold corporate life.

### THE HOUSING SHORTAGE.

That the shortage of housing accommodation is acute is proved by a report of the East Ham Corporation. The Medical Officer of Health states that a large number of applications is being received for lying-in accommodation as a consequence of the lack of housing accommodation in the district. As an emergency measure the Council is endeavouring to make arrangements with the East Ham Cottage Hospital authorities to place beds at the disposal of the Council for this purpose. The Medical Officer has been asked to make inquiries with regard to the rental or purchase of suitable premises in the Borough.

### A MATRON'S RESIGNATION.

Miss Tilson, matron of the Gloucestershire Royal Infirmary, has followed Mr. Pike, the former secretary, into retirement. Thus are partnerships in work and responsibility eventually broken. But no matron, or secretary, need lack for memories and self-congratulation who conducted a hospital safely through the war.

### TRAINING HEALTH VISITORS.

The demand by local authorities for health visitors in connection with maternity and child-welfare work is so great that employment is necessarily being secured for women who are not always well qualified. Meanwhile, steps are being taken to secure the training of women to become health visitors, and so, in the course of time, authorities will be able to insist upon fully-qualified women for such work. The Ministry of Health expects training to be commenced without delay, and, in a circular to councils, hints that after a date which will be fixed later on, grants will be paid only in respect of women who have obtained health-visiting certificates. At East Ham the Medical Officer of Health has suggested that all health visitors who obtain the maternity and child-welfare certificates of the Royal Sanitary Institute should receive an additional £10 per annum, and the Council proposes to adopt this principle.

### A BABY CAMP.

The baby camp which Miss Macmillan established for the benefit of infants and young children under school age at Deptford has proved its value, and the Borough Council now proposes to extend to it official recognition and rate aid to the extent of £300 for one year. The fact that the pioneer work of Miss Macmillan is likely to be extended is suggested in a report of the Health Committee of the Borough Council, which declares that the value of the educational and health work which Miss Macmillan has been doing for many years past in caring for infants and young children under school age is widely recognised, not only so far as the immediate benefit of the children is concerned, but on account of the fact that she is a pioneer in this branch of work, and her activities will have future results of an enduring and far-reaching nature. It is, indeed, reported that the London County Council is arranging to take over the whole of the work in a few years time.

### A CHILDREN'S HOSPITAL FOR BERMONDSEY?

The Mayor of Bermondsey is appealing for £100,000 with which to build a Children's Hospital in Bermondsey as a peace memorial. In The Hospital of April 12 attention was drawn to a project submitted to the local committee by Dr. R. King Brown, the Medical Officer of Health, for acquiring the Princess Club Hospital for wounded soldiers in Jamaica Road, for a children's hospital, but unfortunately the committee was divided in opinion as to the suitability of the premises, and, as the hospital has since been dismantled, the opportunity has gone of obtaining a fully equipped hospital. It is to be hoped that the appeal of the Mayor may be successful, for Bermondsey is very badly off for hospital accommodation for children.

### REFORM BY ARRANGEMENT.

A valuable public health reform has been arranged by negotiation and not by enactment, in the Isle of Wight. The risk of spreading infection during epidemics by the swarming of children to the picture palaces is gradually realised, and the County Council has now been able to persuade all cinema licencees in the island to refuse to admit children if such course is deemed to be advisable by the medical officer of health.

### A SILENT SERVICE.

While the existence of the motor ambulance is accepted, the nature of the work and its demands upon those engaged upon it is little known. It is therefore interesting to read the record of work performed by the Mitcham Division of the St. John Ambulance Brigade. In two and a half months the division, under Sergt. E. Cooper, dealt with fifty-nine cases, two-thirds of which were carried from Mitcham to their homes or to different hospitals in the district, and even in London. It is pointed out that all the above cases required skilled assistance, and that Sergt. Cooper has carefully trained the members of the division. The ambulance staff has dealt with an average of some five or six cases every week, and the calls for its assistance come, of course, at all hours. The St. John Ambulance Brigade has this in common with the Navy—that it is a silent service—but from the above record of one division over a short period the amount of its work and the skill required are evident. Many interesting happenings must occur of which the public knows little, and it is good to have the curtain lifted occasionally.

### AN EIGHTY-FOUR HOUR WEEK.

The Pontypool and District Hospital is unusually fortunate not merely to have begun the past year with a balance in hand, but to have maintained it, and thus starts the current half-year with a balance in hand. Since there are expenses ahead, the Committee hopes that the workers will increase their subscriptions, and since the hospital exists to spend money for the advantage of them and other patients the argument in favour of the proposal is strong. A remarkable statement, however, was made at the recent meeting, when Mr. S. Newman alleged that some of the staff had been working eighty-four hours a week. In reply, the Chairman, Mr. George Jenkins, said that the hours of the staff had been dealt with during the past six months, and were now giving satisfaction. But Mr. Newman should have been asked to be precise, if misunderstanding is to be dispelled.

### THIS WEEK'S DRUG MARKET.

The improved tone noted a week ago is maintained, and a fair amount of business has been transacted. Menthol has attracted attention and the price has advanced. Camphor is also again dearer. Following on a further rise in the price of cloves, quotations for clove oil have been advanced. Star aniseed oil is dearer, but the market is now quieter. The value of potassium bromide is firmly maintained. Essence of lemon is quieter, and the price tendency is inclined downwards. Following on another reduction in the price of quicksilver, quotations for mercurials have again been reduced. Turkey opium has an upward price tendency, but Persian is lower. There is no change in the position of quinine.

